# Commentary: Vitamin C and Metabolic Syndrome: A Meta-Analysis of Observational Studies

**DOI:** 10.3389/fnut.2021.810716

**Published:** 2021-12-14

**Authors:** Kirsten Brandt

**Affiliations:** Human Nutrition Research Centre, Population Health Sciences Institute, Newcastle University, Newcastle upon Tyne, United Kingdom

**Keywords:** bioactive, vegetables and fruits, mortality, phytochemicals, nitrate, folate

## Introduction

The article Guo et al. (1) is a systematic review that was published in Frontiers in Nutrition on 8th October 2021 in the Research Topic “Functional Foods and Bioactive Food Ingredients in Prevention and Alleviation of Metabolic Syndrome.”

The introduction to the review explained the importance of dietary vegetables and fruits for maintaining and enhancing human health, and that intake of these foods is negatively associated with the incidence of the metabolic syndrome (2). Considering that vegetables and fruits are the primary dietary sources of vitamin C, and the review found a significant negative association between vitamin C intake or plasma concentration with incidence of metabolic syndrome, the results of the review clearly confirmed this association. Vegetables and fruits contain a wide range of constituents with potential impact on human health. However, the discussion focused exclusively on the potential role of vitamin C itself, with no mention of any of the other potentially beneficial constituents of vegetables and fruits, such as nitrate (3), phytochemicals (4), and folate (5). There is no mention of the possibility that the vitamin C intake/plasma concentration might be simply a marker of intake of vegetables and fruits, in which case vitamin C itself may have only a minor role in the association or, maybe, none at all.

On 5th November 2021, this article was featured by https://www.nutraingredients.com/, a newsletter for the functional food and nutritional supplements industry, possibly due to perceived relevance to marketing of commercial vitamin C supplements.

## Discussion

None of the abovementioned constituents of vegetables and fruits have been conclusively demonstrated to prevent or alleviate the metabolic syndrome in randomized placebo-controlled trials. To date, the strongest evidence of a causal effect seems to be for folate *via* Mendelian randomization, utilizing genetic variants affecting the bioavailability and metabolism of folate (5). However, as shown by the mostly inconclusive placebo-controlled trials of vitamin C referred to in the article, the evidence for causality of vitamin C is equally weak as for any of the other vegetable and fruit constituents. Additionally, the article fails to refer to the important meta-analysis of mortality caused by vitamin C supplementation, by Bjelakovic et al. (6) or similar literature. This meta-analysis showed that, among 29 intervention trials with low risk of bias, 3,637 of 36,659 vitamin C-supplemented participants died (9.92%), while the mortality among 29,283 placebo-treated participants was only 9.28% (2,717 deaths). This corresponded to a non-significant relative risk of 1.02 (95% CI, 0.98–1.07); however, lack of significance of the evidence of harm is not justification to completely ignore a trend of this magnitude (7% increases in mortality). In fact, a simple Chi square test of these data provides a *P*-value of 0.005 in support of the hypothesis that vitamin C supplementation increases mortality compared with placebo. So, even if future experimental studies confirm that increased vitamin C intake does have beneficial effects on the metabolic syndrome, this must still be presented in the context of the much stronger evidence for harmful side effects of vitamin C supplementation. This caveat is particularly important for researchers planning future intervention trials, as well as for companies considering to use studies like this one in their marketing of vitamin C supplements.

It is critically important for nutrition science to determine which ones of the many correlated and/or confounded dietary constituents, which are significantly associated with health outcomes, actually affect human health, and which ones are primarily confounders or just markers of intake. Once this has been sufficiently demonstrated, it will become relevant to recommend specific foods and/or supplements, which can then be expected to provide the corresponding health benefits. However, until such evidence becomes available, it is essential to focus on the promotion of healthy foods (such as vegetables and fruits) and avoid unjustified encouragement of any potentially harmful nutrient supplementation to individuals not at specific risk of deficiency (7).

In their discussion of the results, Gou et al. repeatedly mention vitamin C deficiency as a risk factor, and they also mention inhomogeneity among the studies. It is clear from the Supplementary Material that the mean intake of vitamin C varied substantially among study populations, and that some of these populations were at a level where vitamin C deficiency is rare or non-existent. The authors are, therefore, encouraged to carry out a subgroup analysis using relevant categories of mean vitamin C intake to investigate whether the observational association was confined to populations at risk of vitamin C deficiency or (also) occurred in such fully replete populations. With this moderate effort, they will be able to assess whether the focus on vitamin C (deficiency) is justified or not, or whether results of the study should be reinterpreted as support for the benefits of intake of vitamin C-rich vegetables and fruits, in general, without implying any particular active constituent in those foods.

## Author Contributions

The author confirms being the sole contributor of this work and has approved it for publication.

## Funding

This work was funded by Biotechnology and Biological Sciences Research Council (BBSRC) in United Kingdom (grant number BBSRC/DH/SA/2019/4).

## Conflict of Interest

The author declares that the research was conducted in the absence of any commercial or financial relationships that could be construed as a potential conflict of interest.

## Publisher's Note

All claims expressed in this article are solely those of the authors and do not necessarily represent those of their affiliated organizations, or those of the publisher, the editors and the reviewers. Any product that may be evaluated in this article, or claim that may be made by its manufacturer, is not guaranteed or endorsed by the publisher.
